# Strengthening regional commitment to ensuring access to medical abortion medicines in WHO’s South-East Asia region: report of a participatory assessment and workshop

**DOI:** 10.1186/s12978-024-01791-4

**Published:** 2024-05-17

**Authors:** Meera Thapa Upadhyay, Terence Fusire, Ulrika Rehnström Loi, Annik Sorhaindo, Mohammed Salahuddin, Mohammed Ayub Hossain, Tashi Tshomo, Erna Mulati, Lovely Daisy, Dian Putri Anggraweni, Tumiur Gultom, Fitri Indrawati, Mariyam Jenyfa, Myint Myint Than, Bharat Bhattarai, Loshan Moonasinghe, Chaminda Mathota, Anchalee Jitruknatee, Celeste Cham, Bela Ganatra, Neena Raina

**Affiliations:** 1 World Health Organization, South-East Asia Region - WHO SEARO, New Delhi, India; 2 Development and Research Training in Human Reproduction (HRP), Department of Sexual and Reproductive Health and Research, UNDP-UNFPA-UNICEF-WHO-World Bank Special Programme of Research, World Health Organization, 20 Avenue Appia, 1211 Geneva, Switzerland; 3 Directorate General of Drug Administration (DGDA) Dhaka, Dhaka, Bangladesh; 4 Ministry of Health, Thimpu, Bhutan; 5 Nutrition, Maternal, Newborn and Child Health, Ministry of Health, Jakarta, Indonesia; 6 Sub Directorate of Drug Safety and Efficacy, Jakarta, Indonesia; 7 Directorate of Drug, Narcotic, Psychotropic, Precursor Distribution and Service Control, Jakarta, Indonesia; 8 Directorate of Safety, Quality and Export-Import of Drug, Narcotic, Psychotropic, Precursor, Addictive Substance Control, Jakarta, Indonesia; 9 Health Protection Agency Ministry of Health, Male, Maldives; 10 Department of Public Health, Ministry of Health, Naypyidaw, Myanmar; 11 Department of Drug Administration Ministry of Health & Population, Kathmandu, Nepal; 12 Family Health Bureau, Ministry of Health, Colombo, Sri Lanka; 13 National Drug Policy Unit, Medicines Regulation Division, FDA, Nonthaburi, Thailand; 14 National Directorate of Pharmacy Ministry of Health, Dili, Timor-Leste

**Keywords:** Access model, Comprehensive abortion care, Medical abortion, National essential medical list, Medical abortion medicines, WHO South-East Asia Region

## Abstract

**Background:**

In 2019, the World Health Organization identified improving access to safe abortion as an important priority toward improving sexual and reproductive health and rights and achieving Sustainable Development Goals. One strategy for addressing this priority is strengthening access to medicines for medical abortion. All 11 countries in the South-East Asia Region have some indications for legal abortion and permit post-abortion care. Therefore, strengthening access to medical abortion medicines is a reasonable strategy for improving access to safe abortion for the Region.

**Methodology:**

We applied an adapted version of an existing World Health Organization landscape assessment protocol for the availability of medical abortion medicines at the country-level in the South-East Asia Region. We collected publicly available data on the existence of national health laws, policies, and standard treatment guidelines; inclusion of medical abortion medicines in the national essential medicines list; and marketing authorization status for medical abortion medicines for each country and verified by Ministries of health. The findings were once more presented, discussed and recommendations were formulated during regional technical consultation workshop. Each country teams participated in the process, and subsequently, the suggestions were validated by representatives from Ministries of Health..

**Results:**

Few countries in the Region currently have national policies and guidelines for comprehensive safe abortion. However, either mifepristone-misoprostol in combination or misoprostol alone (for other indications) is included in national essential medicines lists in all countries except Indonesia and Sri Lanka. Few countries earmark specific public funds for procuring and distributing medical abortion commodities. In countries where abortion is legal, the private sector and NGOs support access to medical abortion information and medicines. Several countries only allow registered medical practitioners or specialists to administer medical abortion.

**Conclusion:**

Following this rapid participatory assessment and technical consultation workshop, the World Health Organization South-East Asia Regional Technical Advisory and Sexual and Reproductive Health and Rights technical committee recommended priority actions for policy and advocacy, service delivery, and monitoring and evaluation, and indicated areas for support.

## Plain Language summary

The World Health Organization suggests improving access to safe abortion to reduce maternal deaths. All 11 countries in the Region permit abortion for some indication. A safe and effective way to end a pregnancy is medical management of abortion with mifepristone and misoprostol pills, or just misoprostol. We reviewed global, regional, and national policy documents, guidelines, training materials and data about the use of medical abortion medicines in South-East Asia and then confirmed the results in a technical consultation workshop with key stakeholders from each country in the Region. We found that about half of the countries had national policies and guidelines related to safe abortion care. Misoprostol is included in the national Essential Medicines Lists in all countries except Indonesia and Sri Lanka. There is no standardized forecasting of resources needed for medical abortion medicines within health budgets in any of the countries except India and Nepal. Most countries only allow registered medical practitioners or specialists to provide medicines for medical abortion. To improve access the medical abortion medicines in the Region the World Health Organization recommended specific actions that should take priority.

## Background

The rate of unintended pregnancy in South-East Asia declined by 21% between 1990–1994 and 2015–2019. During the same period, the abortion rate increased by 21% [1]. Further, the proportion of unintended pregnancies ending in abortion in the region increased from 42 to 65% [1]. Abortion is responsible for approximately 7% of maternal deaths in South-East Asia [2]. In 2019, World Health Organization’s (WHO) South-East Asia Regional Technical Advisory Group (SEAR-TAG) and sexual and reproductive health and rights (SRHR) technical committee identified improving access to safe abortion as an important priority for the region toward improving SRHR and achieving Sustainable Development Goals (SDGs). Among the recommended strategies for addressing this priority was strengthening access to medicines for medical abortion (MA).

Although the context for abortion varies in the 11 countries of the Region, all countries have some indications for legal abortion and permit post-abortion care. All 11 countries in the WHO South-East Asia Region (SEAR)—Bangladesh, Bhutan, Democratic People’s Republic of Korea (DPR Korea), India, Indonesia, Maldives, Myanmar, Nepal, Sri Lanka, Thailand, and Timor-Leste – legally permit abortion to save a woman’s life [3, 4]. Bhutan, India, Maldives and Thailand additionally allow abortion for a wider range of indications, such as preserving a woman’s physical or mental health, in case the pregnancy is a result of rape or incest, or for fetal impairment or abnormality. Nepal has the most liberal indications for abortion in the Region permitting abortion upon request up to 12 weeks' gestation. Bangladesh has provisions for menstrual regulation and post-abortion care during first trimester of pregnancy [4]. Therefore, strengthening access to MA medicines is a reasonable strategy for improving access to safe abortion for all the countries in the Region.

MA, the termination of pregnancy using medication, is a method of abortion recommended by the WHO [5, 6]. The WHO suggests that MA medicines, mifepristone, and misoprostol, be included in National Essential Medicines Lists (NEMLs), registered, marketed, and made available in private, public, and non-governmental sectors in countries according to the specific context. Indeed in 2005, MA medicines were placed on the WHO complementary model list and later, in 2019, added to the core list of the WHO Model List of Essential Medicines [7]. To strengthen access to MA medicines, understanding the existing regional context of their availability is critical.

As part of an overall effort to improve access to all medicines in the Region and toward a health systems approach to expanding and upscaling access to MA, we undertook a rapid participatory assessment and technical consultation workshop to understand the existing availability of MA medicines in the SEA Region. The SEAR-TAG suggested broad stakeholder engagement and data triangulation among ministries, national professional associations, and the WHO to assure regional commitment and robustness of the evidence. Our rapid participatory assessment and technical consultation workshop was also designed to promote stakeholder dialogue and to identify priorities for action in the SEA Region. This paper reports on the process and findings of the rapid participatory assessment and technical consultation workshop on the availability of MA medicines in the SEA Region.

## Methods

We applied an adapted version of an existing WHO landscape assessment protocol for the availability of MA medicines at the country-level in the SEA Region [8]. The original WHO country assessment protocol includes five steps: 1) adaptation of an availability framework, 2) a desk review including a literature review and online data gathering, 3) country-level key informant interviews, 4) analysis of the data to identify barriers and opportunities to improve MA availability, and 5) validation of findings by a technical advisory group. This protocol was previously used in an assessment of eight countries conducted by the UNDP-UNFPA-UNICEF-WHO-World Bank Special Programme of Research, Development and Research Training in Human Reproduction in 2020 [9]. However, given that the aim of this assessment in the SEA Region was to inform a regional dialogue rather than to conduct in-depth national assessments, a technical advisory group was convened, including the Family, Gender Life Course and Essential Drug and Medicine Unit in the WHO SEA Regional office, to adapt the methodology.

For the SEA Region, the adapted protocol relied on a desk review of publicly available data that was later verified by in-country stakeholders and a regional technical consultation workshop to validate and discuss the findings and develop priorities for actions. Given the variability of available data for countries in the Region, the technical advisory group decided to collect data on a small selection of reliable key indicators for MA medicines. These were: availability of national health laws, policies and standard treatment guidelines; inclusion in the NEML; and marketing authorization status.

As part of the desk review, we searched trustworthy publicly available databases, such as the Global Abortion Policies Database [4] and the MEDAB Medical Abortion Commodities Database [10], and reviewed appropriate documents, such as NEMLs, and national policy and procurement guidelines to develop an overview of MA access and availability for each of the 11 countries in the Region. Data collection took place between April and July 2020. For each country, the information gathered in the desk review was compiled into a structured document (Appendix 1) and shared with program managers in the relevant WHO country office for verification. WHO country office program managers further shared the documents with local Ministries of Health (MOHs). Overall, twenty-eight program managers and national professional officers in the Reproductive Health and Drug Administration units in MOHs across the Region verified and provided additional information about availability of and access to MA in country.

Findings from the desk review and responses to the structured documents from each country were compiled, analyzed manually, and presented at a regional technical consultation workshop in November 2020. Approximately 40 stakeholders engaged in family planning and reproductive health, maternal health, supply chain management and drug regulation from MoHs and National Drug Regulatory Agencies in the 11 SEA Region countries, in collaboration with Essential Drug and Medicine and Health System team from the WHO SEA Regional Office, participated in the virtual meeting to validate the assessment findings and agree upon recommendations. Below, we describe the findings from the desk review and the discussions and recommendations arising from the technical consultation workshop.

## Results

We received responses to the structured document from eight of the 11 countries (Bangladesh, Bhutan, Indonesia, Maldives, Myanmar, Nepal, Sri Lanka, and Timor-Leste), and a further two countries provided responses during the regional technical consultation workshop (India and Thailand). The WHO Country Office for DPR Korea provided information for the desk review.

### National health laws, policies, and standard treatment guidelines

Our desk review of national reproductive health policies, standard treatment guidelines for safe abortion care and post abortion care, and training manuals for safe abortion or post abortion care for each country revealed that it was often difficult to access these documents or determine whether these documents existed, particularly in restrictive legal contexts.

Not all countries in the Region currently have comprehensive national policies and guidelines in place for safe abortion (Table 1). Comprehensive abortion care guidelines for safe abortion care existed in Bangladesh, DPR Korea, India, Maldives, Nepal, and Thailand. Bhutan, Indonesia, Myanmar, and Sri Lanka only had guidelines for post-abortion care. Maldives and Timor-Leste did not have either a safe abortion guideline or a post-abortion care guideline (Table 1).

**Table 1:** Inclusion of MA in national policies and guidelines related to reproductive health

Country	Reproductive Health policy/strategy/guideline	Guideline on safe abortion care	Guideline on Post Abortion Care	Training manuals
Bangladesh	**Not included**	**included**	**Not included**	**Not included**
Bhutan	**Not included**	**Not included**	**included**	**Not included**
DPR Korea	**Not included**	**Included**	**Not included**	**Included**
India	**Included**	**Included**	**Not included**	**included**
Indonesia	**Not included**	**Not included**	**included**	**Not included**
Maldives	**Not included**	**included**	**included**	**Not included**
Myanmar	**Not included**	**Not included**	**included**	**Not included**
Nepal	**included**	**included**	**Not included**	**Not included**
Sri Lanka	**Not included**	**Not included**	**included**	**Not included**
Thailand	**Not included**	**included**	**Not included**	**Not included**
Timor Leste	**included**	**Not included**	**Not included**	**Not included**

Stakeholders participating in the technical consultation workshop supported the finding of availability of national guidelines for comprehensive abortion care that are evidence based, regularly updated, and provide the necessary guidance to achieve equitable access to quality care. Where guidelines, policies and/or strategies exist, stakeholders agree that increasing awareness among relevant government agencies, providers and the public is critical to increasing access and minimizing the use of unsafe methods. Where the legal context is restrictive, an enabling policy and regulatory environment is necessary for ensuring that women who are legally eligible have access to safe abortion care and MA medicines.

### Inclusion in national essential medicines lists

We collected information on the inclusion of mifepristone, misoprostol, and the combi-pack (mifepristone 200 mg + misoprostol 200mcg) in the NEMLs of each SEA Region country. For misoprostol, we reported on all formulations, including the 100-mcg formulation which is used for other obstetric indications, such as prevention or management of post-partum hemorrhage among others. Therefore, our analysis includes misoprostol formulations that may not be indicated for use in MA (Table 2).

**Table 2:** National EML status of MA medicines in SEA Region

Country	Mifepristone 200mg	Misoprostol 200mcg/100mcg	Combi-pack (Mifepristone 200mg + Misoprostol 200mcg)
Bangladesh (2016)	**✔**	**✔**	**✔**
Bhutan (2018)	**x**	**✔**	**x**
DPR Korea (2019)	**x**	**✔**	**x**
India (2015)	**✔**	**✔**	**x**
Indonesia (2019)	**x**	**Application under review**	**x**
Maldives (2018)	**✔**	**✔**	**x**
Myanmar (2016)	**x**	✔	**x**
Nepal (2016)	**x**	**✔**	**✔**
Sri Lanka (2014)	**x**	**x**	**x**
Thailand (2019)	**x**	**x**	**✔**
Timor Leste (2015)	**x**	**✔**	**x**

Included in NEML **✔** Not included in NEML **x**

Although mifepristone and misoprostol have been included in the WHO Model List of Essential Medicines for several years [7], of the 11 SEA Region countries, only Bangladesh has included mifepristone, misoprostol, and the combi-pack in its NEML. In addition to either mifepristone and misoprostol, the combi-pack is also included in the NEMLs of only Nepal, and Thailand. None of the medicines have been included in the NEML for Indonesia, although at the time of the study, misoprostol 200 mcg and 100 mcg were under review for inclusion. The remaining countries in the SEA Region have one or more of the compositions included in their NEML (Table 2).

### Marketing authorization status

**Fig. 1:**
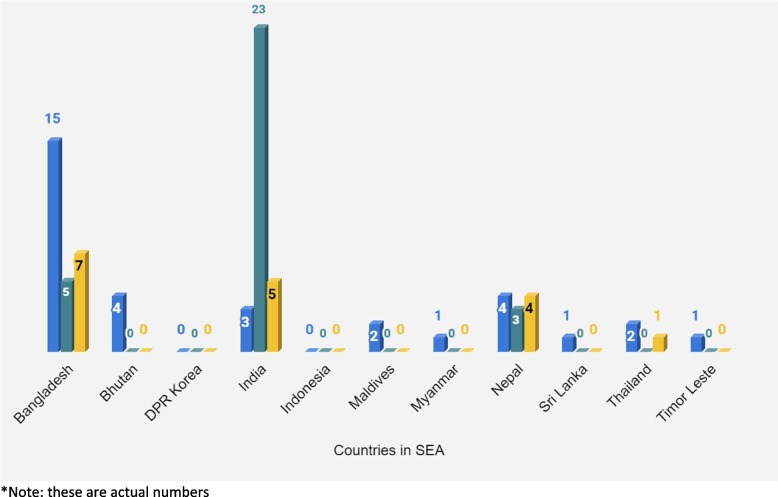
Number of market authorized MA medicines in countries of the Southeast Asia Region. *Note: these are actual numbers. Legend: Misoprostol 100/200mcg (Blue colour in the graph). Mifepristone 200 mg (Green colour in the graph). Combi pack (Mifepristone 200 mg + Misoprostol 200mcg) (Yellow colour in the graph)

Figure 1 illustrates the number of MA products that have been granted approval and registered for market in each of the SEA Region countries. Bangladesh, India, and Nepal have the greatest number of MA products authorized for the market (Fig. 1). Among the remaining countries, DPR Korea and Indonesia have not granted market authorization for any of the essential MA medicines to be used within their respective jurisdictions. In five other countries, namely Bhutan, Maldives, Myanmar, Sri Lanka, and Timor-Leste, the regulatory authority has granted market authorization solely to misoprostol. Stakeholders attending the technical consultation workshop highlighted that in addition to the lack of availability of legally registered products, many countries struggle with the challenges associated with presence of falsified and illegally supplied MA medicines and require increased regulation.

### Financing, procurement, and distribution

In conjunction with the varied market authorization status of MA medicines, countries have adopted diverse approaches to public financing, procurement, and distribution needed to ensure the broad accessibility of these medicines to the general public. In Bangladesh there is no public financing, procurement, or distribution of medicines for MA. However, it is noteworthy that misoprostol is procured for alternative obstetric indications. Similarly in Bhutan, there is no public procurement of MA medicines, apart from misoprostol, which is procured for alternative purposes. Although there is no specific budget for MA medicines in DPR Korea, misoprostol, for other uses, is centrally procured and distributed to government hospitals. In India, states procure and distribute MA medicines, as part of the National Health Mission budget, to hospitals and registered health facilities at the sub-district level. There is no public budget, procurement, or distribution of MA medicines. In Maldives, misoprostol is procured centrally and distributed in designated government hospitals. Misoprostol is procured for active management of third stage of labor and prevention of postpartum hemorrhage through the government annual budget in Myanmar. There is no specific budget or financing for MA medicines in the country, but misoprostol is widely available. In Nepal, funding for MA medicines is decentralized to municipal level health facilities. Medicines are stocked and dispensed at approved health facilities. There is no public budget or procurement for MA medicines in Sri Lanka. In Thailand, there is centralized procurement of MA by specific public hospitals. In Timor Leste, there is no public procurement of MA medicines.

Stakeholders during the workshop reported that there is no standardized forecasting of service requirements and specific provisions for MA medicines within health budgets in any of the countries except India and Nepal. The consultation additionally revealed that there was no incentive in the countries in the Region to support the private sector to expand distribution.

### Access models used across countries

Although MA regimens are safe and can be managed by a broad range of health workers at primary care level, in the SEA Region, who is authorized to provide services is heavily regulated, limiting access. In most countries, registered medical practitioners and/or specialists, such as obstetrician-gynecologists, with specific experience and training can provide MA, as is the case for India, Bangladesh, Myanmar, and Sri Lanka. Nepal allows skilled birth attendants and auxiliary nurse midwives to provide MA after undergoing abortion care training and being certified as MA providers. India, Nepal and Thailand permit pharmacies to dispense MA with a prescription, while Bhutan allows trained nurses to conduct post abortion care in addition to medical practitioners and/or specialists.

In India, Nepal, and Thailand private sector and non-governmental organizations (NGOs) also support access to MA information and medicines. Availability of MA medicines in private pharmacies is very low across the Region, except in a few countries such as Nepal and India. Despite restrictions, some NGOs are also working towards providing improved access to MA both directly and indirectly. Participants in the technical consultation workshop recommended that, for countries such as Bangladesh, Bhutan and Timor Leste, existing strong social marketing organizations and NGO networks should be leveraged to expand access to information and services.

To expand access to points of care, key stakeholders recommended advocacy to increase awareness of national guidelines on safe abortion, the availability of MA medicines, regular training, and mandated certification for service providers. They furthermore suggested widening the geographic coverage of provision to include all public facilities and hospitals, as in the cases of DPR Korea and Thailand, and urban low-income settlements, rural areas and underserved areas through pharmacies and a broader range of health care workers, for example, in India.

Countries in the Region can also learn from successful access models in other countries. For example, in India, Nepal, and Thailand the private sector and NGOs support access to MA information and medicines. In India, 91% of MA occurs outside of facilities [9, 11]. In Maldives MA is fully covered by the social insurance scheme. Another example of an innovative approach to expanding access is the inclusion of the menstrual regulation program in the family planning service in Bangladesh. Other countries in the Region can learn from these models.

## Discussion

This rapid participatory assessment and technical consultation workshop was designed as a first step toward strengthening regional commitment to ensuring access to MA in South-East Asia. However, our approach was somewhat limited. First, the adapted version of the WHO landscape assessment protocol for availability of MA medicines used in this rapid assessment did not include key informant interviews and did not allow for the collection of in-depth information at the country-level. We relied exclusively on publicly available data and information from brief interactions with stakeholders in the technical consultation workshop. Nevertheless, this rapid assessment provides a coherent overview of the availability of MA medicines in the SEA Region and ignited active engagement of relevant stakeholders in data collection, validation and in determining priority strategies.

Overall, we found that legal abortion in the Region is mixed and, in many cases, quite limited. Such legal restrictions hinder access for people seeking services and act to fuel abortion stigma [12]. Restrictive legal environments have also been strongly associated with an increase in unsafe abortions and maternal mortality in low-middle-income countries [13]. Improved awareness of existing policies, guidelines and strategies is important to promote safe abortion care in SEA Region. Stakeholders participating in the technical consultation workshop added that this variation in the existence of abortion laws, policies and standard treatment guidelines across countries has resulted in limited access, poor quality of care and persistent abortion stigma in the Region.

All countries in the Region permit MA for legal abortion but few include medicines designated for MA in their NEMLs. The countries with liberal legal provisions—India, Nepal, and Bangladesh—have a greater number of quality-assured MA medicines authorized for the market. Registration of multiple sources of products for each medicine will ensure sustainable access [9]. In the technical consultation workshop, stakeholders were clear that advocacy efforts should be directed towards inclusion of all MA medicines in the NEMLs of all the countries in the SEA Region.

Even though MA medicines are low-risk, simple to administer and can be safely managed by health workers [5, 14] some countries continue to impose restrictions that create significant barriers to access. Task sharing, and the potential use of telemedicine is recommended to improve access to comprehensive abortion care services including MA. For the public sector, MA medicines should be accessible to non-physician providers, such as nurses and midwives as per the guidelines [5, 14]. Similarly, for the private sector, countries may consider access through certified pharmacists where regulation allows.

Stakeholders attending the technical consultation workshop participated in a live poll to put forth recommendations and potential strategies based upon the assessment findings. The results of the live poll showed that 89% of participants thought that advocacy for policies to support the availability and use of MA within the country’s abortion legal context were required for improving access to MA medicines in the country. About 40 percent of stakeholders in the technical consultation workshop thought that encouraging market authorization of a greater number of products was essential for improving access to MA medicines in the Region. Close to half (44%) of participants supported strengthening procurement and logistics for MA medicines. Furthermore, a third suggested enhancing budgeting and financing for MA medicines. They also recommended that MOHs centrally forecast, budget specifically for, procure and distribute all MA medicines for permitted uses.

Forty-one percent of stakeholders in the technical consultation workshop believed that all approved MA medicines should be included in the NEMLs of countries in the Region regardless of the legal context. For example, the inclusion of MA medicines, mifepristone, and misoprostol, in the NEML of the Maldives was identified as an example of a strategic and innovative approach to advancing access to safe abortion and MA in a restrictive context. At the technical consultation workshop, 44% of stakeholders desired expansion of point of care access. Where policy support exists, wider distribution should follow. SEA Region countries tend to have extensive networks of health centers, remote health posts, mobile clinics, and community health workers already providing reproductive health services and could add MA to their existing services.

Following this rapid participatory assessment and technical consultation workshop, the SEAR-TAG and SRHR technical committee recommended priority actions for policy and advocacy, service delivery, and monitoring and evaluation, and indicated areas for support. They suggested strengthening engagement with MOHs and professional associations to bolster legal frameworks and policies that improve access to safe abortion for all women and adolescents. They encouraged MOHs in the Region to ensure financial resources, supplies, including MA medicines, competent heath workforce, accurate data systems and public awareness of abortion services, as well as a rights-based approach.

To reinforce service delivery, they suggested that MOHs install program managers dedicated to the delivery of high-quality safe comprehensive abortion care at national and sub-national levels, and further engage with the private sector including pharmacies, and community-based organizations to improve access to services. Finally, the WHO aimed to support countries to include abortion-related indicators in their health information systems and monitor the program against national targets. These actions are ongoing and rely on high-level regional commitment and collaboration among a range of relevant sectors, some of which were established during this rapid participatory assessment and technical consultation workshop.

## Conclusion

This rapid participatory assessment and technical consultation workshop provided a foundation from which to develop effective strategies to expand safe abortion access and the availability of MA medicines in the SEA Region.

## Data Availability

The data that support the findings of this study are available from the corresponding author, MTU, upon reasonable request.

## Abbreviations

MA: Medical Abortion
Mcg: Micro gram
Mg: Milli gram
MOH: Ministry of Health
NEML: National Essential Medical List
NGO: Non-Governmental Organization
SEA: South-East Asia
WHO CO: WHO Country Office

## References

[1] Bearak J, Popinchalk A, Ganatra B, Moller A, Tunçlap Ö, Beavin C, Kwok L, Alkema L (2020). Unintended pregnancy and abortion by income, region, and the legal status of abortion: estimates from a comprehensive model for 1990–2019. Lancet Glob Health.

[2] Say L, Chou D, Gemmill A, Tunçlap Ö, Moller A, Daniels J, Gülmezoglu AM, Temmerman M, Alkema I (2014). Global causes of maternal death: a WHO analysis. The Lancet.

[3] Lavelanet AF, Schlitt S, Johnson BR (2018). Global Abortion Policies Database: a descriptive analysis of the legal categories of lawful abortion. BMC Int Health Hum Rights.

[4] WHO. Global Abortion Policies Database. https://abortion-policies.srhr.org/?mapq=q1h

[5] World Health Organization (2022). Abortion care guideline.

[6] World Health Organization (2018). 2018 Medical Management of Abortion. Department of Reproductive Health and Research.

[7] WHO electronic Essential Medicines List (eEML), World Health Organization, 2022. https://list.essentialmeds.org/. Licence: CC BY 3.0 IGO

[8] Rehnström Loi U, Prata N, Grossman A, Lavelanet A, Williams N, Ganatra B (2023). In-country availability of medical abortion medicines: A description of the framework and methodology of her WHO landscape assessments. Reprod Health..

[9] Grossman A, Prata N, Williams N, Ganatra B, Lavelanet A, Läser L, Asmani C, Elamin H, Ouedraogo L, Rahman MM, Conneh-Duworko MJ, Tehoungue BZ, Chanza H, Phiri H, Bhattarai B, Dhakal NP, Ojo OA, Afolabi K, Kabuteni TJ, Hailu BG, Moses F, Dlamini-Nqeketo S, Zulu T, Rehnström Loi U. Correction: Availability of medical abortion medicines in eight countries: a descriptive analysis of key findings and opportunities. Reprod Health. 2023;20(1):160. 10.1186/s12978-023-01691-z. Erratum for: Reprod Health. 2023;20(Suppl 1):58. 10.1186/s12978-023-01574-3 PMC10091522 37041543

[10] IPPF. International Medical Advisory Panel (IMAP) Statement on medical abortion – Intended to support and guide those providing information and services, engaged in advocacy and/or partnering with government and other key stakeholders. Oct 2018 (https://www.ippf.org/sites/default/files/2019-10/IPPF_IMAP_Statement%20on%20medical%20abortion%20-%20English.pdf)

[11] Singh S, Shekhar C, Acharya R, Moore A, Stillman M, Pradhan MR, Frost J, Sahoo H, Alagarajan M, Hussain R, Sundaram A, Vlassoff M, Kalyanwala S, Browne A (2018). The incidence of abortion and unintended pregnancy in India, 2015. Lancet Glob Health.

[12] Janet M. Turan and Henna Budhwani, 2021: Restrictive Abortion Laws Exacerbate Stigma, Resulting in Harm to Patients and Providers American Journal of Public Health 111, 37_39, 10.2105/AJPH.2020.305998 10.2105/AJPH.2020.305998 PMC7750605 33326286

[13] Ngo NV, Pemunta NV, Basil N, Tembe FE, Eyambe MS, Ezra K, Ngwa HC, Sabo EO (2021). Reproductive health policy Saga: Restrictive abortion laws in low- and middle-income countries (LMICs), unnecessary cause of maternal mortality. Health Care Women Int.

[14] World Health Organization (2015). Health Worker Roles in Providing Safe Abortion Care and Post-abortion Contraception.

